# The histone 3 lysine 4 methyltransferase, Mll2, is only required briefly in development and spermatogenesis

**DOI:** 10.1186/1756-8935-2-5

**Published:** 2009-04-06

**Authors:** Stefan Glaser, Sandra Lubitz, Kate L Loveland, Kazu Ohbo, Lorraine Robb, Frieder Schwenk, Jost Seibler, Daniela Roellig, Andrea Kranz, Konstantinos Anastassiadis, A Francis Stewart

**Affiliations:** 1 Genomics, BioInnovationsZentrum, Technische Universitaet Dresden, Am Tatzberg, 01307 Dresden, Germany; 2 Centre for Regenerative Therapies Dresden, BioInnovationsZentrum, Technische Universitaet Dresden, Am Tatzberg, 01307 Dresden, Germany; 3 Monash Institute of Medical Research, Monash University, Melbourne and ARC Centre of Excellence in Biotechnology and Development, Australia; 4 Department of Histology and Cell Biology, School of Medicine, Yokohama City University, Yokohama, Japan; 5 The Walter and Eliza Hall Institute of Medical Research, Melbourne, Australia; 6 TaconicArtemis Pharmaceuticals GmbH, Neurather Ring, 51063 Cologne, Germany; 7 University of Applied Science Gelsenkirchen, Department of Applied Natural Sciences, August-Schmidt-Ring, 45665 Recklinghausen, Germany

## Abstract

**Background:**

Histone methylation is thought to be central to the epigenetic mechanisms that maintain and confine cellular identity in multi-cellular organisms. To examine epigenetic roles in cellular homeostasis, we conditionally mutated the histone 3 lysine 4 methyltransferase, Mll2, in embryonic stem (ES) cells, during development and in adult mice using tamoxifen-induced Cre recombination.

**Results:**

In ES cells, expression profiling unexpectedly revealed that only one gene, *Magoh2*, is dependent upon Mll2 and few other genes were affected. Loss of Mll2 caused loss of H3K4me3 at the *Magoh2 *promoter and concomitant gain of H3K27me3 and DNA methylation. Hence Mll2, which is orthologous to *Drosophila *Trithorax, is required to prevent Polycomb-Group repression of the *Magoh2 *promoter, and repression is further accompanied by DNA methylation. Early loss of Mll2 *in utero *recapitulated the embryonic lethality found in *Mll2*-/- embryos. However, loss of Mll2 after E11.5 produced mice without notable pathologies. Hence Mll2 is not required for late development, stem cells or homeostasis in somatic cell types. However it is required in the germ cell lineage. Spermatogenesis was lost upon removal of Mll2, although spermatogonia A persisted.

**Conclusion:**

These data suggest a bimodal recruit and maintain model whereby Mll2 is required to establish certain epigenetic decisions during differentiation, which are then maintained by redundant mechanisms. We also suggest that these mechanisms relate to the epigenetic maintenance of CpG island promoters.

## Background

Epigenetic mechanisms play several roles in genome function including the maintenance of genome stability, suppression of transposons, dosage compensation and imprinting, as well as the regulation of gene expression. Despite much progress, the understanding of gene regulation by epigenetic mechanisms remains far from complete. For example, epigenetic mechanisms contribute to the differences in gene expression patterns between somatic cells, and to the loss of pluripotency during development [1-4]. However, it is still not understood whether these mechanisms direct decisions during lineage commitment and differentiation, or merely secure them.

Amongst the several classes of histone post-translational modifications, the six lysine methylations on histones 3 and 4 have emerged as central to epigenetic mechanisms, with each apparently relating to a different chromatin status [3]. In mammals, unraveling epigenetic roles in gene expression is likely to prove difficult. Even assuming that epigenetic mechanisms are largely confined to DNA and histone methylation, the existence of multiple enzymes for each modification complicates the analysis. For example, by bioinformatic criteria, there are at least six H3K4 and seven H3K9 methyltransferases amongst more than 42 candidate enzymes [5]. Of these, at least half appear to be pairs of sister genes, indicating that redundancy may obscure studies to determine their functional roles. Nevertheless, knock-outs of six of the nine histone methyltransferases studied so far are embryonic lethal in the mouse [5-12].

The studies reported here began with the aim to explore and compare epigenetic functions in pluripotent and somatic cells. We reasoned that epigenetic mechanisms secure a particular cellular state by maintaining a characteristic gene expression pattern. Thereby a single genome can be reliably utilized in many different ways because epigenetic modifications fix the different gene expression patterns that define different cellular states [2-4,13]. Consequently a role for epigenetics in cellular homeostasis is implied.

**Figure 1:**
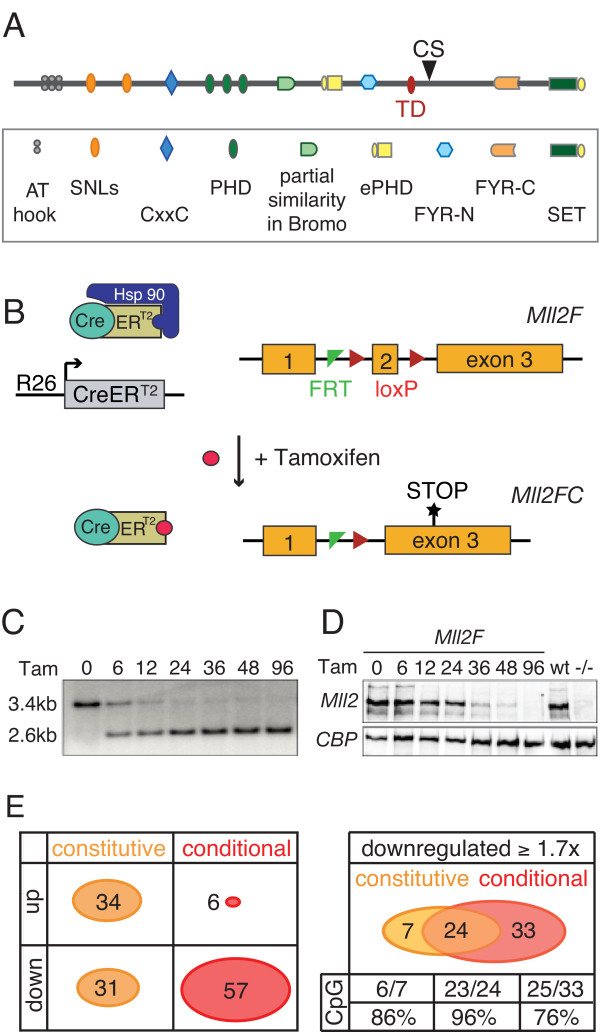
**Conditional Mutagenesis of *Mll2***. (A) Scheme of the Mll2 protein, which contains several domains and motifs including AT hooks; SNLs (speckled nuclear localization sequences); the CxxC DNA binding region; three PHD fingers and an extended PHD finger (ePHD); a sequence similar to a Bromo domain; the FYR-N and -C domains (which dimerize); a transactivation domain (TD); a cleavage site (CS) for Taspase; and the SET domain, which is the H3K4 methyltransferase domain. (B) Scheme of the strategy for conditional mutagenesis. The CreERT2 protein was ubiquitously expressed from the *Rosa26 *locus but repressed by the Hsp90 complex. Tamoxifen binding releases CreERT2 from Hsp90 to permit Cre recombination of the loxP sites surrounding exon 2 of *Mll2*, which causes a frame-shift mutation. (C) A Southern blot to determine recombination efficiency in *Mll2F/F; Rosa26-CreERT2/+ *ES cells at various timepoints (0 to 96 hours) after addition of 4-hydroxy tamoxifen. (D) The same time course as shown in (C) was evaluated for Mll2 protein levels by western blotting. Wild type (wt) and *Mll2-/- *ES cells served as controls. (E) Summary of microarray expression profiling from *Mll2-/- *(constitutive) cells compared with wt or FLP rescued cells and tamoxifen-treated (conditional) compared with untreated *mll2F/F; Rosa26-CreERT2/+ *cells. At the left, the summary shows the number of genes whose mRNA expression level increased or decreased at least 1.7-fold in either the constitutive or conditional experiment. At the right, the overlap between the down-regulated genes in the two experiments is shown, along with the number of CpG island promoters in the three categories.

To explore these issues, we chose the H3K4 methyltransferase Mll2/Wbp7 (Figure 1A), which is the sister of Mll (mixed lineage leukemia) by gene duplication [14]. Both *Mll *and *Mll2 *are widely expressed throughout development and in adults and both are required for embryogenesis, however show different phenotypes when knocked-out [5,7,12]. Hence exploration of their cellular and somatic roles requires a strategy for conditional mutagenesis. We used ligand-regulated site-specific recombination [15] mediated by Cre recombinase-mutant estrogen receptor fusion proteins (Cre-ERT) to induce loss of *Mll2*. We aimed at temporal recombination without spatial restrictions by using a *Cre-ERT2 *transgene [16] targeted to the ubiquitous *Rosa26 *locus [17]. Thereby functional enquiry can be directed towards any cell at a chosen time, which is a prerequisite for the study of homeostasis.

## Results

### Tamoxifen induces complete loss of Mll2

Using a multi-purpose 'knock-out first' allele [18] for *Mll2*, we previously found that *Mll2 *null embryos die before E10.5 [5]. To assess Mll2 function in embryonic stem (ES) cells, after E10.5 and in adult mice, we converted the knock-out allele into a conditional one by FLPe-mediated removal of the stop cassette [19]. The conditional allele (termed '*F*' for FLP recombined) includes loxP sites flanking the 73 bp second exon. Removal of exon 2 by Cre recombination invokes a frame shift in the mRNA resulting in a premature stop codon and a knock-out allele termed '*FC*' (Figure 1B). Both *Mll2*-/- and *Mll2FC/FC *embryos show the same developmental lethal phenotype [5]. We made ES cells from *Mll2F/F; Rosa26-CreERT2/+ *blastocysts. 4-hydroxy tamoxifen induction resulted in over 95% recombination by 24 hours (Figure 1C) and loss of Mll2 protein after 48 hours (Figure 1D).

To determine candidate Mll2 target genes in ES cells, we harvested RNA for Affymetrix expression profiling before and after 96 hours of tamoxifen induction to yield 'conditional' samples (Figure 1E). For greater accuracy, we also harvested RNA from our previously described double-targeted *Mll2*-/- and FLP rescued ES cell lines [20] to yield 'constitutive' samples. A summary of expression changes greater than ~1.7 fold (*p *values <0.0001) is shown in Figure 1E. The constitutively mutated cells showed a similar number of up and down-regulated genes at this threshold (34 and 31 respectively). However, the conditionally mutated cells showed 10-fold greater down-regulated genes than up (57 and 6, respectively), which is consistent with expectations from the published roles of Mll2 as a transcriptional activator and/or maintenance factor. Of the six up-regulated genes in the conditional experiment, four (*Ly6a, Tbx3, 1700097N02Rik, 2210409E12Rik*) were also found amongst the 34 constitutively up-regulated genes. In contrast to this discordance of up-regulated genes, the two experimental approaches show significantly better agreement for the down-regulated genes (Figure 1E). In addition to slightly different genetic backgrounds for the two ES cell lines (129 versus mixed 129/C57Bl6), we suggest that the different results are due to secondary adaptations in the constitutively mutated cells. These cells were derived from a single cell after targeting followed by more than 25 cell cycles in culture before mRNA was harvested. In contrast, the conditionally mutagenized cells lost Mll2 within the previous 72 hours *en masse *and so the expression changes will more directly reflect Mll2 action. Notably most of the down-regulated genes in the conditional experiment are expressed from CpG island promoters (48/57), particularly in the group that overlapped with the constitutive experiment (23/24), which are most likely to be directly regulated by Mll2.

### Magoh2 is a direct target for Mll2

**Figure 2:**
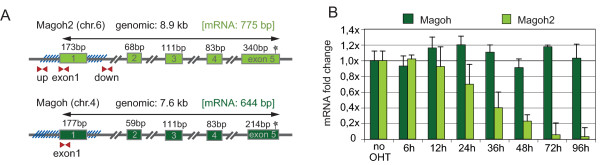
**The *Magoh2 *promoter is a direct target for Mll2 and a model example of *trxG*/PcG opposition (part 1)**. (A) Diagrams of the *Magoh2 *and *Magoh *genes, showing the exon structures, the positions of the CpG island promoters (as hatched lines around exon 1) and the PCR primer pairs for chromatin immunoprecipitation (ChIP) analysis. (B) Histogram of quantitative RT-PCR analysis of *Magoh *(dark green) and *Magoh2 *(light green) mRNA levels before and after addition of 4-hydroxy tamoxifen to *Mll2F/F; Rosa26-CreERT2/+ *cells.

The 10 most down-regulated genes in common between the two experimental approaches were evaluated by quantitative reverse transcriptase polyermase chain reaction (RT-PCR) and good agreement to the microarray data was found for all of them (Additional file 1). Given the published roles of the H3K4 methyltransferases in transcriptional activation and/or maintenance, we were surprised to find very few genes down-regulated more than about twofold in absence of Mll2 and only one gene whose expression completely relied upon Mll2. This gene is a duplication of *Magoh *and was previously unnamed (2010012C16Rik). Hence we call it *Magoh2 *(Figure 2; Additional file 2). Befitting the role of Magoh as a housekeeping function in mRNA export [21], both *Magoh *and *Magoh2 *appear to be ubiquitously expressed in all tissues examined (Additional file 2B).

**Figure 3:**
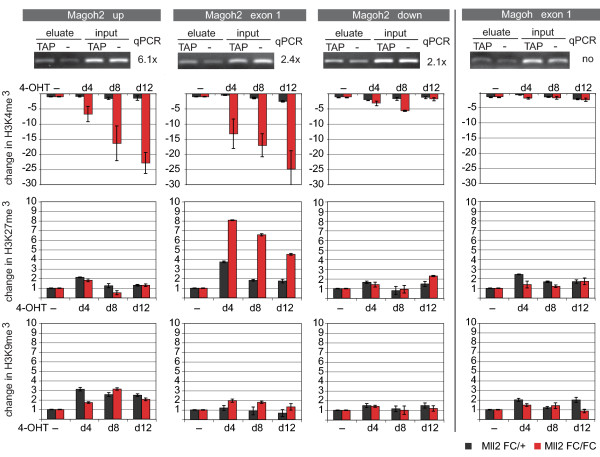
**The *Magoh2 *promoter is a direct target for Mll2 and a model example of *trxG*/PcG opposition (part 2)**. ChIP analysis of the *Magoh2 *and *Magoh *promoters, as indicated at the top for the *Magoh2 *up, exon 1, down and *Magoh *exon 1 Q-PCR primer pairs as diagrammed in (Figure 2A). The first row of panels shows ChIP results obtained from TAP-tagged Mll2 ES cells using the TAP tag for immunoprecipitation compared with 5% of the input before immunoprecipitation. Foldness of Mll2-specific enrichment is noted at the side of each of the four panels. The second, third and fourth row of panels show fold change of chromatin immunoprecipitation using H3K4me3, H3K9me3 and H3K27me3-specific antibodies. *Mll2F/F; Rosa26-CreERT2/+ *(Mll2FC/FC)*or Mll2F/+; Rosa26-CreERT2/+ *(Mll2FC/+) cells were cultured for 12 days without (-) or with 4 days of 4-hydroxy tamoxifen for the last 4 days (d4), the middle 4 days (d8) or the first 4 days (d12).

After tamoxifen induction, *Magoh2 *mRNA levels fell (Figure 2B) in concordance with falling Mll2 protein levels (Figure 1D), whereas *Magoh *mRNA levels were unchanged. Similarly, Mll2 occupancy and H3K4me3 levels fell at the *Magoh2 *but not *Magoh *promoter (Figure 3). For technical reasons, the loss of Mll2 at the *Magoh2 *promoter was difficult to detect. However, the loss of H3K4me3 was greater than 20-fold, both immediately 5' to, and within, its CpG island promoter.

We next looked for changes in the opposing histone methylation marks at H3K27 and H3K9. Within the CpG island of *Magoh2*, H3K27me3 levels increased more than sixfold, whereas H3K9me3 levels did not change. Notably, H3K27me3 levels also went up slightly in the heterozygous control cells (*Mll2F/+; Rosa26-CreERT2/+)*. This suggests that the balance of H3K4me3 to H3K27me3 within the CpG island was also responsive to a 50% reduction of Mll2 protein level. No other H3K27me3 (or K9me3) changes were apparent, including immediately outside of the CpG island on both 5' and 3' sides, even though losses of H3K4me3 occurred on the 5' side.

**Figure 4:**
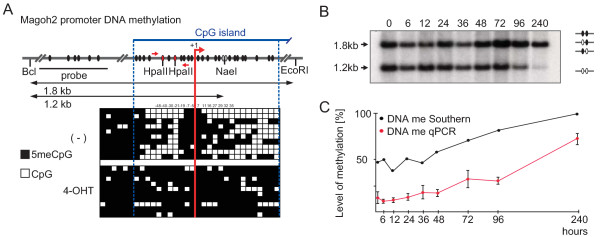
**The *Magoh2 *promoter is a direct target for Mll2 and a model example of *trxG*/PcG opposition (part 3)**. (A) Bisulfite sequencing results of the *Magoh2 *promoter before (-) and after (4-OHT) addition of tamoxifen for 4 days, followed by a further 8 days of culture shown below a diagram of the promoter, which illustrates the location of the CpG dinucleotides as well as the relevant restriction sites, Southern probe and PCR primers (small arrows either side of the Hpa1 sites) for the results shown in panels (B) and (C). (B) Southern blot to evaluate CpG methylation at the *Nae*I site of the *Magoh2 *promoter as illustrated in panel (A). Because the *Nae*I site contains two CpGs, resistance to restriction can reflect methylation at either or both sites, as illustrated at the right of the panel. The time course was performed on *Mll2F/F; Rosa26-CreERT)/+ *cells starting with addition of 4-hydroxy tamoxifen. (C) As for panel (B) except the genomic DNA samples were digested with *Hpa*I before Q-PCR analysis with the primer pair illustrated in panel (A). These data are plotted (red circles, lower plot), as well as quantitation of the Southern blot shown in panel (B; black circles, upper plot).

As evaluated by bisulfite sequencing (Figure 4A), CpG methylation-sensitive Southern blotting (Figure 4B and 4C) or PCR (Figure 4C), the *Magoh2 *promoter became CpG methylated following the loss of Mll2 protein and H3K4me3. These results show that *Magoh2 *requires Mll2 for expression in ES cells, in part due to the antagonism of repression mediated via H3K27 and CpG methylations.

### Mll2 is only required in a brief developmental window and not in adult mice

**Figure 5:**
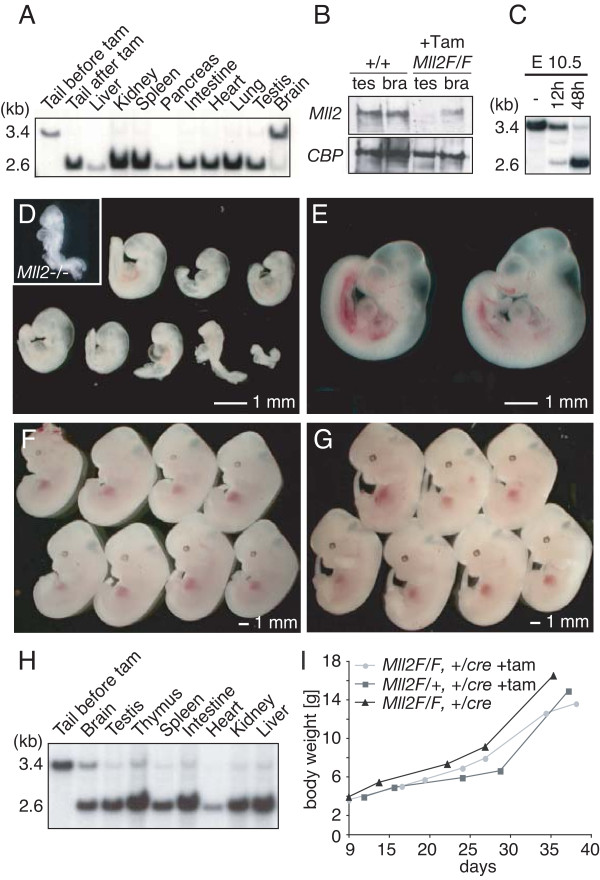
**Ligand-induced mutagenesis of *Mll2 in utero*, via lactation and in adults**. (A) Southern blot of tamoxifen-induced CreERT2 recombination in various tissues of a 2-month-old *Mll2F/F; Rosa26-CreERT2/+ *male two weeks after tamoxifen administration. The first lane shows tail DNA before tamoxifen administration with the 3.4 kb unrecombined band. All other lanes show DNAs after tamoxifen displaying the recombined 2.6 kb band. (B) Western blot of protein extracts from wild type and tamoxifen induced *Mll2F/F; Rosa26-CreERT2/+ *male testis and brain. The 300 kD protein, CBP, was used as a loading control. (C) Recombination efficiency in E10.5 embryos was detected by Southern blot. Pregnant mothers were untreated (-) or treated with one dose of 4 mg tamoxifen at E10 (12 h) or E8.5 (48 h). (D) The *Mll2*-/- phenotype was recapitulated in *Mll2F/F; Rosa26-CreERT2/+ *embryos after induction at E4.5 and E5.5 with doses of 1 mg tamoxifen. The embryos were harvested at E10.5. The inset shows a *Mll2-/- *embryo at E10.5 at the same scale. (E) Control *Mll2F/+; Rosa26-CreERT2/+ *embryos induced with tamoxifen at E4.5/E5.5 were normal at E10.5. (F, G) Mll2 is dispensable from E11.5. *Mll2F/F; Rosa26-CreERT2/+ *(F) and *Mll2F/+; Rosa26-CreERT2/+ *embryos (G) induced at E8.5 were normal at E12.5. (H) Southern blot of various tissues harvested from 14-day-old *Mll2F/F; Rosa26-CreERT2/+ *neonates, using the same strategy as in panel (A) and five daily doses of 4 mg tamoxifen to the lactating mothers, starting 4 days after delivery. (I) Body weight measurements after birth of *Mll2F/F; Rosa26-CreERT2/+ or Mll2F/+; Rosa26-CreERT2/+ *pups treated with tamoxifen or not as in panel (H).

Though Mll2 is only required for a small amount of mRNA expression in ES cells, an *Mll2 *knock-out was lethal before E10.5 [5]. To investigate whether Mll2 is required after E10.5, particularly for somatic homeostasis, tamoxifen was used to induce Cre recombination in 2-month-old adults. Although we could not induce efficient recombination in the brain, recombination elsewhere was nearly complete (Figure 5A and 5B). Unexpectedly we found that adult mice lacking Mll2 appeared normal. These mice showed only slight abnormalities, had normal weight and blood profiles, lived as long as their littermates and were not prone to tumorigenesis or any other notable pathology (data not shown). As ligand-induced recombination will never be absolutely complete [15], we checked old *Mll2FC/FC; Rosa26-CreERT2/+ *mice for repopulation of various compartments with the unrecombined allele, particularly in blood. No repopulation was observed under any circumstance (data not shown), indicating that Mll2 is dispensable for most somatic stem cells and cell types.

In the course of these experiments, which include more than 200 FC/FC mice so far, we discovered that tamoxifen treatment of homozygous *Rosa26-CreERT2 *mice caused death in more than half of the cases due to an intestinal complication, which includes diarrhea, altered bile secretion, stomach distension and an apparent loss of peristalsis (Additional file 3). This problem required tamoxifen administration and homozygosity of *Rosa26-CreERT2 *but not the *Mll2 *conditional (*F*) allele (either heterozygous or homozygous). We avoided the problem by performing tamoxifen inductions in *Rosa26-CreERT2/+ *heterozygotes.

To determine when Mll2 was required, we administered tamoxifen to both pregnant and lactating mothers to induce recombination *in utero *or in neonates, respectively. Recombination was induced *in utero *by administration of tamoxifen to pregnant *Mll2F/F *females after crossing to *Mll2F/F; Rosa26-CreERT2/Rosa26-CreERT2 *homozygous males. For control litters, the same female genotype was crossed with *Mll2+/+; Rosa26-CreERT2/Rosa26-CreERT2 *homozygous males to produce *Mll2F/+; Rosa26-CreERT2/+ *embryos. Pregnant females received either two doses of 1 mg tamoxifen on two consecutive days (E4.5/5.5; E8.5/9.5) or one dose of 4 mg (E8.5 and later) by oral gavage. Recombination was virtually complete in all mutant embryos within 48 hours (Figure 5C and data not shown). Induction at E4.5/5.5 provoked the same embryonic lethal phenotype observed in *Mll2 *null embryos, namely pleiotropic growth and developmental retardations with death before E10.5 (Figure 5D). Tamoxifen administration at E8.5/9.5 showed no obvious differences between *Mll2FC/FC *or *Mll2FC/+ *littermate controls at E12 (Figure 5F and 5G). Pups induced at E12.5/13.5, whether control or *Mll2FC/FC*, were normal at birth but died due to poor fostering by the tamoxifen-treated mothers, as apparent from failures to clean away the placenta or to suckle and lactate. Untreated foster mothers rescued both control and *Mll2 *deleted pups (data not shown). Poor fostering was not observed when tamoxifen was administered to the mothers after birth.

Five doses of 4 mg tamoxifen administered by gavage once a day from P5 to P9 induced near-complete recombination in the pups, indicating that tamoxifen is efficiently transmitted through the milk by suckling (Figure 5H). All tamoxifen-treated pups were monitored for 6 weeks and developed normally. Tamoxifen-induced recombination in the neonatal brain was more efficient than in adults but still incomplete. Whereas control pups not receiving tamoxifen gained weight more rapidly than tamoxifen-treated pups, there was little difference between *Mll2FC/FC *and *Mll2FC/+ *pups (Figure 5I). This indicates that tamoxifen administration has a minor effect on growth of neonates, probably by reducing lactation of the mother [22], while the absence of Mll2 does not. Taken together with the results from tamoxifen treatment of 2-month-old adults, we conclude that Mll2 is only essential before E10.5 in development and is not required for further development or somatic homeostasis. If loss of Mll2 protein *in utero *upon tamoxifen induction shows the same kinetics as it does in ES cells (Figure 1D), we can narrow this window to between E7.5 and E10.5.

### Male mice lacking Mll2 are infertile

**Figure 6:**
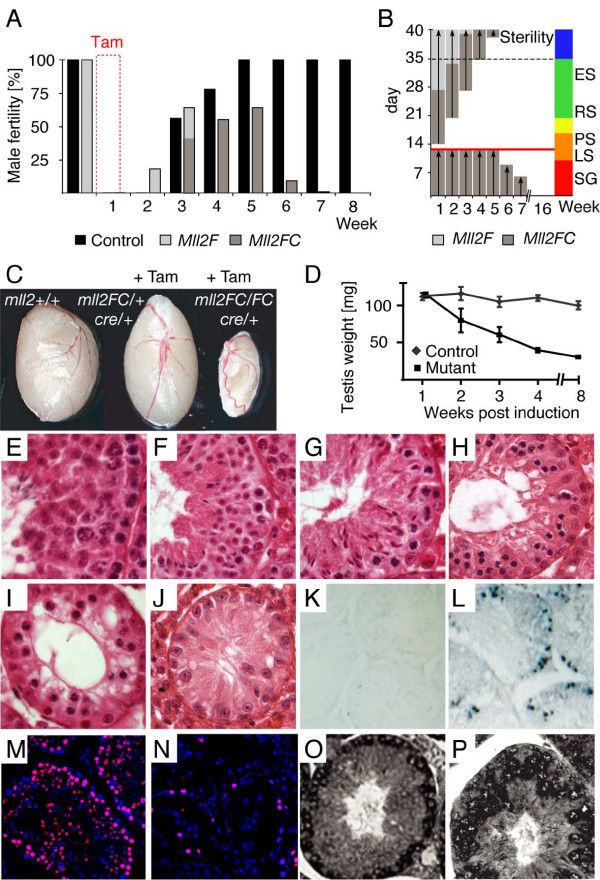
**Loss of Mll2 leads to male sterility**. (A) The fertility of 11 control (black) or mutant males (grey) was tested by weekly breedings to two wild-type females before and after tamoxifen administration. The mutants initially transmitted the unrecombined haplotype (light grey), and then the recombined haplotype (dark grey) and lost fertility by week 7. (B) The diagram illustrates a spermatogenic period of 40 days with mitosis (red), meiosis I (orange), meiosis II (yellow), spermiogenesis (green) and spermatozoa maturation (blue). Indicated are spermatogonia (SG), leptotene spermatocytes (LS), pachytene spematocytes (PS), rounded spermatids (RS) and elongated spermatids (ES). The transmission of the unrecombined haplotype (light grey) indicates that recombination did not occur in ES and only partially occurred in RS. Although recombination was complete in LS and PS, spermatogenesis proceeded (dark grey). Permanent sterility occurring in the sixth week indicated that spermatogenesis was interrupted before or at meiosis I (red line) (C, D) Wild-type, control and atrophic mutant testes 8 weeks after tamoxifen induction (C) or weighed during a time course (D). (E to J) Testis histological cross-sections reveal a block of germ cell differentiation and progressive loss of spermatogonia in *Mll2FC/FC *testis. Control testis (E) were normal after tamoxifen treatment. Mutant testis were sectioned 1 week (F), 2 weeks (G), 3 weeks (H), 4 weeks (I) and 14 weeks (J) after induction. (K, L) Increased levels of spermatogonial apoptosis in *Mll2FC/FC *testis, determined by TUNEL staining in control (K) and mutant (L) testis sections 3 weeks after tamoxifen induction. (M, N) Persistence of Tra98-positive cells 4 months after tamoxifen induction. Double staining with a Tra98 antibody and DAPI shows the expected distribution of Tra98 in control testis (M) and reduced staining in mutant testis (N). (O, P) Expression of *Mll2 *(O) and *Mll *(P) in normal testis detected by *in situ *hybridization with antisense probes.

Although we did not find any somatic abnormalities after tamoxifen treatment, *Mll2FC/FC; Rosa26-CreERT2/+ *males and females became infertile. To investigate the male infertility, we established breeding experiments. First, 11 male (Figure 6A and 6B) *Mll2F/F; Rosa26-CreERT2/+ *(mutant) or *mll2F/+; Rosa26-CreERT2/+ *(control) mice were individually caged with two female wt mice each. All mice gave successful fertilizations. The males were then treated with tamoxifen followed by introduction and weekly replacement of two wt females to establish the time courses shown in Figure 6A and 6B. Both control and mutants showed infertility in the first and second weeks after tamoxifen treatment, followed by recovery in the third and fourth weeks (Figure 6A). In the control males, fertility was fully recovered by the fifth week. However, recovery in the *FC/FC *males was transient and complete infertility was observed after the sixth week.

Genotyping the offspring revealed that the mutant males transmitted the unrecombined haplotype (*Mll2F*) in the second and third weeks and the recombined haplotype (*Mll2FC*) from the third to sixth weeks. As spermatogenesis in the mouse takes 5 weeks with 5 days of epididymal transition (Figure 6B) these results permit the following conclusions:

1. As loss of Mll2 did not prevent fertility between weeks 3 and 6, male infertility is due to failures in spermatogenesis and not because of the loss of other abilities. This conclusion is supported by the observations that serum testosterone levels and plugging frequencies were normal in *Mll2FC/FC *males (data not shown).

2. As the unrecombined allele was transmitted in weeks 2 and 3, recombination was inefficient in the late stages of spermatogenesis (elongated spermatids and maturing spermatozoa).

3. The transmission of both haplotypes (*Mll2F *and *Mll2FC*) in the third week shows partial recombination in round spermatids.

4. Sole transmission of the recombined haplotype 4 to 6 weeks after tamoxifen treatment indicates that complete recombination occurred in primary and secondary spermatocytes and spermatogonia.

5. Sterility in the sixth week after induction indicates that the interruption of spermatogenesis occurred very early during the spermatogonial stages.

### Male infertility is caused by a block of spermatogenic differentiation

The mutant testes were obviously smaller after 2 months (Figure 6C and 6D). Histological examination confirmed the progressive germ cell loss inferred in Figure 6B. The earliest visible abnormality was a dramatic reduction in spermatocytes 2 weeks after induction (Figure 6F). Whereas Sertoli and interstitial cells, spermatogonia and preleptotene spermatocytes (stages VII–VIII) appeared normal, we observed reduced frequencies of leptotene/zygotene spermatocytes (stages IX–XII) and early pachytene spermatocytes (stages I–IV). These observations, together with the absence of degenerating spermatocytes, indicated a developmental block in spermatogenic differentiation before the early pachytene stage. Three weeks after tamoxifen induction, the tubules contained spermatogonia, preleptotene spermatocytes and spermatids but no pachytene spermatocytes (Figure 6G). In addition to the immediate arrest of germ cell differentiation we also observed a progressive depletion of spermatogonia at later stages (Figure 6H to 6J). Increased apoptosis of spermatogonia was detected from 1 week after induction and peaked around 3 weeks (Figure 6K and 6L). Staining for Tra98 4 months after tamoxifen induction showed the presence of Tra98-positive cells, indicating that some spermatogonia persisted (Figure 6M and 6N).

These observations indicate one or two primary defects in *Mll2FC/FC *testes: (i) a developmental block in spermatogenic differentiation before the pachytene stage; (ii) a defect in the production of spermatogonial B cells from A cells.

The *Mll2 *expression pattern in testes concords with a role for Mll2 in spermatogonia because *in situ *hybridization revealed strong expression in these cells, but weak or absent expression in spermatocytes, Sertoli and Leydig cells (Figure 6O). Interestingly, we observed a complementary *Mll *expression pattern with strong signals in spermatocytes and Leydig cells, and a weak or absent signal in spermatogonia (Figure 6P).

### Molecular characterization of Mll2FC/FC testes

**Figure 7:**
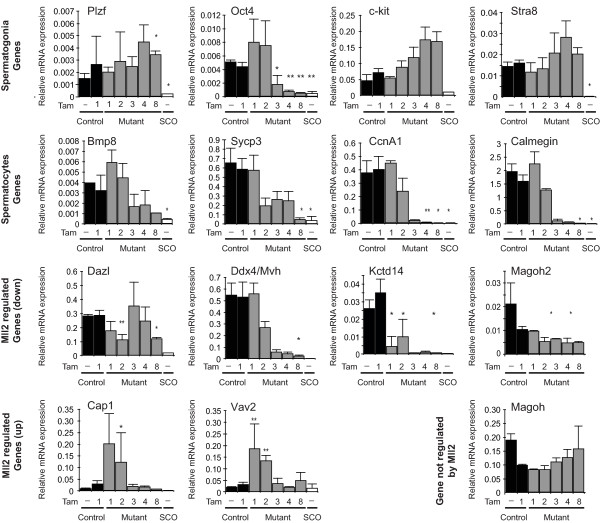
**Expression analysis by quantitative RT-PCR of selected genes in *mll2 *mutant and control testes**. The expression levels from total testes of controls (black) before (-) or 1 week after tamoxifen were compared with mutants (grey) after tamoxifen administration. All times refer to weeks after the start of five daily doses of 4 mg tamoxifen. Busulfan-treated testes were used to generate Sertoli-cell only controls (SCO). All data is based on at least three mice. The mean value ± SEM of relative expression normalized against the endogenous standard genes GAPDH and *Rpl19 *is shown (* *P *< 0.05; ** *P *< 0.02).

Quantitative RT-PCR analysis on total testis RNA at several time points after tamoxifen induction (1, 2, 3, 4 and 8 weeks) was used to characterize alterations in gene expression (Figure 7). As a first control, we compared gene expression in uninduced control males with control males 1 week after tamoxifen treatment. No significant differences were detected, indicating that tamoxifen did not influence expression of these genes (Figure 7). As a second control, RNA was also harvested from testes treated with busulfan, which clears the germ lineage leaving only Sertoli and Leydig cells. As markers for spermatogonia, we examined expression of *Plzf*, *C-kit *and *Stra8 *[23-25]. Expression of these genes persisted, which concords with the observed persistence of Tra98-positive cells (Figure 6N). In contrast, *Oct4*, which is normally co-expressed with *Plzf *in undifferentiated spermatogonia [26], was strongly reduced 3 weeks after induction, suggesting a gene-specific effect of *Mll2 *mutagenesis.

Next we tested genes that are expressed in spermatocytes at prepachytene (*Bmp8, Sycp3*) and pachytene (*CcnA1, Calmegin*) stages [27]. These genes all showed decreased expression over the time course in agreement with the increasing depletion of post-spermatogonial stages seen in the histology. Whereas *CcnA1 *and *Calmegin *expression disappeared rapidly, *Sycp3 *and *Bmp8 *expression initially decreased but persisted for several weeks before completely disappearing. This indicates that pachytene spermatocytes were rapidly lost, while some leptotene and zygotene spermatocytes persisted. We also looked at a panel of Sertoli-specific genes, which showed continued expression as expected, except for *rhox3 *and *rhox11 *(Additional file 4A). Notably both of these genes were also depleted by busulfan treatment, suggesting that their expression is dependent upon signals received by the Sertoli cells from spermatogenesis.

To identify genes with altered expression after loss of Mll2 but before the cellular composition of the mutant testis was affected, expression profiling was performed by microarray analysis from testes 6 days after tamoxifen induction (Additional file 4C). Major candidates were verified by quantitative RT-PCR, including the candidate with the most reduced expression, *Kctd14*, and two candidates that were dramatically up-regulated, *Cap1 *and *Vav2 *(Figure 7). We also evaluated testis expression of three Mll2 target genes identified in ES cells, namely *Magoh2, Ddx4 *and *Dazl*. *Ddx4 *expression in testis was lost with similar kinetics as *Oct4*, suggesting a loss of expression in spermatogonia. *Dazl *and *Magoh2 *showed a twofold reduction in expression (Figure 7). For *Dazl*, sustained expression in testis obviously does not require Mll2 and is concordant with the persistence of spermatogonia A [28]. For *Magoh2*, the twofold reduced expression in testis upon loss of Mll2 stands in stark contrast with the complete reliance on Mll2 observed in ES cells (Figures 2, 3 and 4).

## Discussion

Epigenetics was first conceived as a necessary mechanism for multi-cellular organisms to use a single genome in multiple ways [4,13]. Roles for epigenetic mechanisms in transcriptional regulation, defining or confining cellular identity and maintaining homeostasis follow from this concept. The work described here started with the intention to look for epigenetic roles in the maintenance of cellular identity by use of conditional mutagenesis to remove an H3K4 methyltransferase, Mll2, from somatic states. Unexpectedly, we found that many cellular identities are apparently indifferent to the removal of Mll2. However, Mll2 is required during a developmental window and in both male and female germ lineages. We discuss the implications of these findings in turn.

### Redundancy of mammalian H3K4 methyltransferases

Mammals have at least six H3K4 methyltransferases in three pairs of sister genes [5]. Consequently functional redundancy, or overlapping functional plasticity and compensation, is plausible. Nevertheless, because Mll2 is essential during development, we did not expect it to be thoroughly dispensable in adult mice (except possibly in the brain, which we could not evaluate because tamoxifen-induced recombination was incomplete). Previously we established that Mll2 could be removed from ES cells by gene targeting of both alleles. However, a number of defects were observed, including an increased level of apoptosis due to decreased *Bcl2 *expression and a strongly decreased ability to differentiate towards the neural lineages [20]. Hence significant somatic perturbations were expected in the adult mouse upon removal of Mll2. In contrast, our extensive investigations have only revealed subtle somatic effects so far (SG, AK, unpublished observations).

Removal of Mll2 by conditional mutagenesis in ES cells permitted an accurate appraisal of changes in mRNA levels and also revealed only subtle alterations. Only one gene, *Magoh2*, was found to rely entirely on Mll2 for expression and only a few were altered more than twofold. Because these slight changes do not explain the ES cell phenotype, we think that mRNA profiling has been insufficient and other circuitry, such as microRNA expression, should be evaluated.

Regulation of *Magoh2 *by Mll2 is an illuminating case study. Like all validated Mll2 target genes, *Magoh2 *is expressed from a CpG island promoter. Mll2 and its sister, Mll, contain the CxxC protein domain, which binds unmethylated CpGs [29-31]. This domain is also present in Cfp1, which is a subunit of the mammalian Set1A and Set1B H3K4 methyltransferase complexes [32,33]. As unmethylated CpGs are normally only found in CpG islands [34,35], potentially the CxxC domain localizes these four H3K4 methyltransferases to CpG island promoters. For technical reasons, we have been unable to explore this proposition by global ChIP, either using Mll or Mll2-specific antibodies, or Mll and Mll2-tagged proteins. However a ChIP-on-chip analysis in U937 cells for Mll found an almost complete concordance of Mll ChIP with Pol II and active genes [36], which could reflect a level of redundancy within the H3K4me3 system whereby any H3K4me3 promoter, once established, will recruit one or more of the other six H3K4 methyltransferases, potentially via PHD fingers either within the enzymes themselves (for Mll1–4) or the complex Cfp1/Spp1 (for the Set1A and B complexes; [37]). Hence, it may be difficult to establish the non-redundant relationships between promoters and H3K4 methyltransferases.

In ES cells, expression of *Magoh2 *collapses upon removal of Mll2. Loss of Mll2 is accompanied by loss of H3K4me3 but also gain of H3K27me3 at the *Magoh2 *promoter. Hence the Trithorax orthologue, Mll2, is required to oppose repression mediated, in part, by a Polycomb-Group mechanism. This example concords with the opposition between *trx-G *and Pc-G action as elucidated in flies [1,38]. Furthermore, we observe that CpG methylation adds an extra level of repression to Pc-G repression without the apparent involvement of H3K9 methylation. Hence, as known for X-chromosomal inactivation [39], Pc-G repression can be accompanied by CpG methylation also on an autosomal gene. This has important implications for the epigenetic causes of tumorigenesis, which have hitherto focused on the acquisition of tumor suppressor gene silencing [40]. We suggest that mechanisms which provoke loss of *trx-G *maintenance of tumor suppressor gene expression should also be examined carefully.

### Mll2 is only required during a brief developmental window

**Figure 8:**
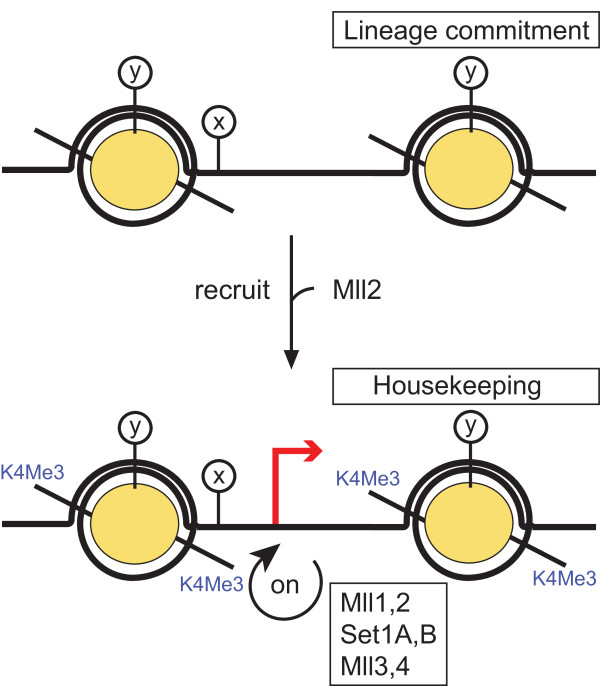
**The recruit and maintain (RAM) model to explain the transient requirement for Mll2**. The diagram illustrates two nucleosomes at a gene promoter, to which Mll2 is recruited. Once recruited, Mll2 mediates the establishment of a back-up system, partly via H3K4 trimethylation, which is based on its sister, Mll, and potentially the other H3K4 methyltransferase pairs, Set1A/Set1B and Mll3/Mll4. The epitopes, x and y, are included to indicate that other chromatin determinants are likely to play roles in recruitment and maintenance.

Conditional mutagenesis allowed us to identify the period in which Mll2 is essential. This period encompasses gastrulation and early organogenesis. Two questions arise. Why is Mll2 essential during this period? And why is it not essential at other times? To answer these questions, we propose the following bimodal explanation, which we call the 'recruit and maintain' (RAM) model. First, in almost all cellular states, Mll2 is part of an implicitly redundant or overlapping mechanism(s), which includes one or more of the other H3K4 methyltransferases (Mll, Set1A, Set1B, Mll3, Mll4). Second, during lineage commitment in gastrulation and organogenesis, new patterns of gene expression are established, which include the lineage-specific selection of certain genes to be epigenetically maintained in the active state. Third, the selection of these genes includes the recruitment of *trx-G *components, such as a H3K4 methyltransferase. Fourth, in certain cases, only one of the H3K4 methyltransferases is recruited and in its absence, none of the others substitute. Fifth, once recruited, the implicitly redundant or overlapping mechanism is established and propagates the activity of the promoter (Figure 8).

Above we pointed out the potential relationship between Mll, Mll2 and CpG island promoters. Virtually all housekeeping genes are expressed from CpG island promoters. However, in contrast to the understanding of TATA box promoters, the understanding of CpG island promoters is thin [41]. We suggest that the underlying basis for the activity of housekeeping promoters in all cells is epigenetic, based in part on an inheritable mechanism involving H3K4me3, Mll1, Mll2, Set1A and Set1B. Furthermore, we suggest that this constitutive cellular mechanism is selectively recruited to certain genes during lineage commitment to convey inheritable (epigenetic) activation in a lineage-specific pattern. Or stated more plainly, these certain genes become like 'housekeeping' genes for the purpose of the specific lineage.

The RAM model offers an explanation for the lack of a major phenotype in conditionally mutated *Mll *mice. Using a *Vav-Cre *line for conditional mutagenesis that ablated Mll expression in the liver and blood lineages, McMahon et al [7] bypassed the embryonic lethality of Mll knock-outs to show that Mll could be removed from these cell types during late development and in the adult without notable impact. Although this analysis was confined to a few cell types, it does concord with the findings we report here for Mll2 and the RAM model.

Notably the *Magoh2 *promoter in ES cells appears to be an exception to the RAM model. It is difficult to explain why the *Magoh2 *promoter in ES cells relies solely on Mll2 and cannot be supported by any of the other H3K4 methyltransferases. This suggests that other determinants in addition to CpG dinucleotides and H3K4me3 are involved in defining CpG island promoters. Alternatively, this may be another unusual aspect of ES cell chromatin [42]. Notably, *Magoh2 *does not rely on Mll2 in testis (Figure 7).

### Mll2 is required in the male and female germ lineages

Both male and female germ lineages require Mll2 at a specific stage, which is likely to be shortly before meiosis in both cases. Loss of Mll2 does not prevent primordial germ cell migration into the genital ridge (SG, AK unpublished observations). The nature of the female germ line defect will be the subject of a different paper. Here we established that the defect in the male germ line occurs between spermatogonia A and the pre-meiotic pachytene spermatocyte stages, resulting in apoptosis and consequently a complete loss of spermatogenic cells. However, the persistence of the spermatogonia A stem cells, as shown by the persistence in mutant testis of various defining mRNAs and Tra98-positive cells, indicates that the defect occurs upon exit from the stem cell state. Although more work needs to be done to define the nature of this defect, we suggest that it also concords with the RAM model. Namely, Mll2 is not required to maintain the spermatonial stem cell but is required upon exit from the stem cell state, presumably due to changes in gene expression. Spermatogenesis may be idiosyncratically susceptible to the loss of Mll2 because Mll is not sufficiently co-expressed in the spermatogonia (Figure 6P). Notably, our data indicate that removal of Mll2 does not eliminate spermatogonia A; rather, the defect is revealed in the committed daughter cells.

### Mll2 target genes

*Ddx4 *and *Dazl *were both high on the list of down-regulated genes from the ES cell expression profiling. Along with Magoh2, the fly homologues of these three proteins are involved in germ cell granules in the germ line [43-45], and loss of either Ddx4 (also called Mouse vasa homologue) and Dazl (deleted in azoospermia), causes male sterility in mice [27,28]. Therefore potentially Mll2 regulation underpins an evolutionarily conserved germ cell program.

Although we did not observe any gene ontology linkages among the candidate Mll2 target genes, a strong correlation with CpG island promoters was observed. So far, 23 of the top 24 Mll2 candidate target genes identified in ES cells have CpG islands (Figure 1; Additional file 1). At the threshold used, approximately 60% of all mouse promoters are CpG islands. As the chance that 23 of 24 are CpG islands at this threshold is less than 1E-7, we find support for a relationship between Mll2 and CpG islands.

### Conditional mutagenesis

Conditional mutagenesis in the mouse has proven to be remarkably powerful since its first application [46] and the addition of ligand regulation [15-17,47-49] has further increased its precision. However, it is not without its problems [50-53] to which we add another one (Additional file 3). Nevertheless, when applied carefully the value of these tools greatly outweighs their deficiencies, which emphasizes the need to design experiments with the proper controls. Here we used the heterozygously floxed allele as the control for the homozygously floxed experiment. In both cases, the *Rosa26-CreERT2 *allele was heterozygously present and tamoxifen induction caused the same Cre recombination either heterozygously for the control or homozygously for the conditional mutation.

Ligand-induced recombination added temporal control to conditional mutagenesis. Its first useful applications in mice were based on a combination of temporal and spatial regulation [47-49]. Here we applied it solely for temporal regulation using expression from the *Rosa26 *locus. The absence of background recombination before induction, and the rapid and near ubiquitous recombination after induction, demonstrate the good properties of this gene switch. However, we did not achieve complete recombination in the brain. We believe this is due to the weak, but not absent, expression from the *Rosa26 *promoter in post-mitotic neurons [54], rather than the inability of tamoxifen to cross the blood-brain barrier.

## Conclusion

The studies reported here began with the intention to examine epigenetic mechanisms in the maintenance of cellular identities using conditional mutagenesis of Mll2 as the model case. Unexpectedly we found that Mll2 is only required briefly in certain conditions. During development it is not required in the pluripotent stem cells but at certain points around gastrulation. During spermatogenesis, it is not required in spermatogonia A stem cells but in the differentiating spermatogonia. We therefore propose a model whereby Mll2 function is only occasionally essential during alterations of cellular gene expression programs and otherwise is supported on established promoters by a redundant mechanism(s). Furthermore, we find a correlation between Mll2 target genes and CpG island promoters and report a case where Mll2 is required to prevent PcG repression of a CpG island promoter. PcG repression is further secured by DNA methylation.

## Methods

### ES cells

Methods with ES cells have been described elsewhere [20,55]. In addition, *Mll2F/F; Rosa26-CreERT2/+ *and *Mll2F/+; Rosa26-CreERT2/+ *ES cell lines were isolated from blastocyst outgrowths. Cre-mediated recombination was induced by 10^-6 ^M 4-hydroxy tamoxifen from 10^-4 ^M ethanol stocks.

Affymetrix gene chip analyses were performed with mouse Set430_A arrays for the constitutive experiment or mouse Set430_2.0 arrays for the conditional experiment. The constitutive experiments were performed in triplicate whereas the conditional experiments were performed in quintuplicate. Conditional *Mll2 *ES cells were grown without feeders on 0.1% gelatine-coated plates, induced for 48 h or not and harvested 96 h after the start of induction. RNA extraction and processing were according to the manufacturers' instructions. Statistical analyses of gene expression profiles were performed with students *t*-test ANOVA. Wild-type and FLP-rescued ES cell gene expression data were paired and compared with constitutive *Mll2*-/- data. Probe sets with a statistical significance (*p*-value) lower than E-04 were used for analysis. Microarray data of conditional ES cells were analyzed by pairwise comparison of genes with *p*-values lower than E-05 in a students *t*-test ANOVA.

Chromatin immunoprecipitation with H3-K4me^3^, H3-K27me^3 ^(Abcam) and H3-K9me^3^-specific antibodies (Upstate) used conditional *Mll2F/F; Rosa26-CreERT2/+ *ES cells after induction with 4-hydroxy tamoxifen for 48 h and crosslinked at day 4, day 8 and day 12. Uninduced ES cells as well as tamoxifen-induced *Mll2F/+; Roasa26-CreERT2/+ *ES cells were used as controls. Immunoprecipitations were carried out as described [56] and analyzed by quantitative PCR analysis.

Primer pairs for bisulfite sequencing were Magoh2_BiS and Magoh2_BiSnested. For Southern blot analysis, a 550 bp genomic probe for the *Magoh2 *promoter was generated by PCR amplification with the primer pair Magoh2_probe. Genomic DNA of tamoxifen-treated or untreated conditional *Mll2F/F *ES cells was digested with *Eco*RI, *Bcl*I and methylation-sensitive *Nae*I. Methylated CpG dinucleotides at positions +32 and/or +35 produced a 1.8 kb fragment with *Nae*I-digested DNA samples, while a 1.2 kb fragment was detected as long as both sites were unmethylated. Methylation at two CpG dinucleotides (positions -48 and -30) overlapping with two *Hpa*II restriction sites was detected by quantitative PCR with primer pair Magoh2_HpaII. Genomic DNA of tamoxifen-treated or untreated conditional *Mll2F/F *ES cells was fragmented with *Rsa*I, which does not cleave between the primer pair and therefore determined the C_t _value of the assay corresponding to uncleaved template. *Rsa*I/*Hpa*II-digested DNA was used to determine the amount of uncleaved template corresponding to methylation at both CpG dinucleotides. *Rsa*I/*Msp*I-digested DNA served as negative control and did not produce a PCR product (primers listed in Additional file 5).

### Mice

The Mll2-/- mice, genotyping and Western methods were described previously [5]. Homozygous *Mll2+/- *were crossed to hACTB-Flpe mice to generate *Mll2F/+*. Intercrosses of *Mll2F/+ *mice produced homozygous *mll2F/F *mice at the expected Mendelian ratio, which were crossed to the *Rosa26-CreERT2 *line. An *Nla*III fragment of *Mll2 *cDNA (321 to 988) was used as probe that hybridized to 3.4 kb or 2.6 kb bands for *Mll2F *and *Mll2FC *respectively.

For *in vivo *tamoxifen inductions, tamoxifen base (Sigma, T5648) was dissolved in peanut oil (Sigma) at 80 mg/ml. The solution was shaken rigorously at 55°C until the tamoxifen was completely dissolved and divided into 50 μl aliquots for storage at -20°C. Mice received one aliquot containing 4 mg tamoxifen per day for five consecutive days. Aliquots were heated at 37°C and given by gavage with a 1.2 mm × 40 mm feeding needle (037-0129 MediQuick).

Testes were fixed for 6 hours in Bouin's fixative, dehydrated in a graded ethanol series, and embedded in paraffin. Mounted sections where used for hematoxylin/eosin stainings, for immunohistochemical staining, and TUNEL assays. For immunohistochemistry, antigen retrieval was performed in 50 mM glycine (ph 3.5; 90°C maintained for 8 min). TUNEL assays were performed on sections treated with Proteinase K (10 μg/ml) for 15 min at 37°C using the In situ Death Detection Kit (Roche). In situ hybridization was performed with digoxigenin-labeled cRNA probes (Mll, bp 11897 to 12400 of Accession no. NM001081049; Mll2, bp 8151 to 8458 of Accession no. NM029274) as previously described [57]. Total RNA from testes were isolated using TriReagent (Sigma), pretreated with Dnase I (Promega) and reverse-transcribed with oligo-dT primers and Superscript III (Invitrogen). DNA samples from at least three individual mice for each time point after induction were analyzed by the SYBR green method using a Stratagene MX4000. All assays produced C_t _values below 30 (Additional file 4B) and were normalized against levels of GAPDH and Rpl19 mRNAs (all primers are listed in Additional file 5).

## Competing interests

The authors declare that they have no competing interests.

## Authors' contributions

SG, SL, KA and AFS conceived and designed the study, and wrote the manuscript. KLL and KO performed testis *in situ *hybridization and immunohistochemistry. LR, FS and JS contributed reagents and materials. SG, AK and KA conducted experiments with mice. SL, SG and DR conducted experiments with embryonic stem cells. All authors have read and approved the manuscript.

## Supplementary Material

Additional File 1 **Expression profiling in embryonic stem cells**. The primary list of down-regulated genes from the Affymetrix experiments. The absence of data in the constitutive columns is due to the fact that these experiments were performed with an earlier version of the mouse array that did not have probes for these genes. The top 10 down-regulated genes found in both constitutive and conditional experiments were evaluated by quantitative RT-PCR. The presence of a CpG island at exon 1 of the respective genes is noted in the right hand column. Click here for file

Additional File 2 **Comparison of *Magoh *and *Magoh2***. (A) The amino acid sequences of Magoh2 and Magoh are aligned. (B) Expression analysis by quantitative RT-PCR was performed for various mouse tissues taken from a 2-month-old male. Click here for file

Additional File 3 **Intestinal complications in *Rosa26-CreERT2 *homozygous mice**. (A, B) An intestinal display of a tamoxifen-treated *Rosa26-CreERT2/+ *heterozygous mouse (A) and of a tamoxifen-treated *Rosa26-CreERT2/Rosa26-CreERT2 *homozygous mouse (B). Both mice were Mll2 +/+ and were dissected 10 days after the start of tamoxifen administration (five daily doses of 4 mg). In panel (B) the bile duct (arrow) is swollen, the stomach and small intestine is distended. Diarrhea and a rectal prolapse were also evident. (C) Sick *Rosa26-CreERT2 *homozygous mice had symptoms of dehydration as indicated by weight loss (data not shown) and increased stomach weights compared with controls. (D) Summary of tamoxifen-provoked deaths in various genotypes. Click here for file

Additional File 4 **Additional expression data from mll2FC/FC; Rosa26 CreER(T2)/+ testes**. (A) As for Figure 7, quantitative RT-PCR of selected genes. (B) Mean C_t _values of quantitative RT-PCR for selected genes on five control (wild-type) testes. (C) Results from Affymetrix analysis of control and mutant testes 6 days after the start of tamoxifen treatment. Only the greatest fold changes, either down (at top) or up (at bottom), are shown, along with *p*-values. The results are the average of two independent experiments. Click here for file

Additional File 5 **Sequences of primers.** Click here for file
